# The cancer-associated cell migration protein TSPAN1 is under control of androgens and its upregulation increases prostate cancer cell migration

**DOI:** 10.1038/s41598-017-05489-5

**Published:** 2017-07-12

**Authors:** Jennifer Munkley, Urszula L. McClurg, Karen E. Livermore, Ingrid Ehrmann, Bridget Knight, Paul Mccullagh, John Mcgrath, Malcolm Crundwell, Lorna W. Harries, Hing Y. Leung, Ian G. Mills, Craig N. Robson, Prabhakar Rajan, David J. Elliott

**Affiliations:** 1 Institute of Genetic Medicine, Newcastle University, Newcastle-upon-Tyne, UK; 2 Northern Institute for Cancer Research, Newcastle University, Newcastle-upon-Tyne, UK; 3 NIHR Exeter Clinical Research Facility, Royal Devon and Exeter NHS Foundation Trust, Exeter, UK; 4 Department of Pathology, Royal Devon and Exeter NHS Foundation Trust, Exeter, UK; 5 Exeter Surgical Health Services Research Unit, Royal Devon and Exeter NHS Foundation Trust, Exeter, UK; 6 Department of Urology, Royal Devon and Exeter NHS Foundation Trust, Exeter, UK; 7 Institute of Biomedical and Clinical Sciences, University of Exeter, Devon, UK; 8 Cancer Research UK Beatson Institute, Glasgow, UK; 9 Institute of Cancer Sciences, University of Glasgow, Glasgow, UK; 10 Prostate Cancer Research Group, Centre for Molecular Medicine Norway (NCMM), Nordic EMBL Partnership, University of Oslo and Oslo University Hospitals, Forskningsparken, Gaustadalléen 21, N-0349 Oslo, Norway; 11 Department of Molecular Oncology, Institute for Cancer Research, Oslo University Hospital HE - Norwegian Radium Hospital, Montebello, Ian G. Mills, NO-0424 Oslo, Norway; 12 Movember/Prostate Cancer UK Centre of Excellence for Prostate Cancer Research, Centre for Cancer Research and Cell Biology (CCRCB), Queen’s University Belfast, 97 Lisburn Road, Belfast, BT9 7AE UK; 13 Barts Cancer Institute, Queen Mary University of London, John Vane Science Centre, Charterhouse Square, London, EC1M 6BQ UK

## Abstract

Cell migration drives cell invasion and metastatic progression in prostate cancer and is a major cause of mortality and morbidity. However the mechanisms driving cell migration in prostate cancer patients are not fully understood. We previously identified the cancer-associated cell migration protein Tetraspanin 1 (TSPAN1) as a clinically relevant androgen regulated target in prostate cancer. Here we find that TSPAN1 is acutely induced by androgens, and is significantly upregulated in prostate cancer relative to both normal prostate tissue and benign prostate hyperplasia (BPH). We also show for the first time, that TSPAN1 expression in prostate cancer cells controls the expression of key proteins involved in cell migration. Stable upregulation of TSPAN1 in both DU145 and PC3 cells significantly increased cell migration and induced the expression of the mesenchymal markers SLUG and ARF6. Our data suggest TSPAN1 is an androgen-driven contributor to cell survival and motility in prostate cancer.

## Introduction

Cancer, in its most aggressive form, is not only a disease of uncontrolled cell growth, but also a disease of inappropriate cell migration. Activating invasion and metastasis is a hallmark of cancer progression^1, 2^ and is the leading cause of mortality among cancer patients^3^. Metastasis involves cancer cells detaching from the primary tumour, and travelling as circulating tumour cells through the bloodstream or lymphatic system to other parts of the body. Prostate cancer is the most common male cancer in Europe, with around 50,000 new cases in the UK each year^4^. At initial diagnosis 37–43% of men have late stage disease and 17–34% of prostate cancer patients have metastasis (http://www.cancerresearchuk.org/health-professional/cancer-statistics/statistics-by-cancer-type/prostate-cancer/incidence#ref-8). The development of prostate cancer is initially driven by androgen steroid hormones via the androgen receptor (AR) transcription factor. The first line treatment for prostate cancer that is no longer organ confined is androgen deprivation therapy (ADT). However, after 2–3 years many patients develop castrate resistant prostate cancer (CRPC) for which treatment options are limited and prognosis is poor^5^, meaning there is an urgent need to develop new treatments for advanced prostate cancer. Prognostic heterogeneity is an important feature of prostate cancer; while some prostate cancers can progress very rapidly, others can remain indolent for many years, hence there also a major unmet clinical need to identify new biomarkers to help distinguish indolent from aggressive disease^6^.

The mechanisms underlying the development and progression of prostate cancer are poorly understood. We recently used RNA-Sequencing to comprehensively profile how the prostate cancer transcriptome responds to androgens^7^. Our approach directly correlated gene expression data from LNCaP cells before and after androgen exposure, with data from a small cohort of 7 prostate cancer patients before and after ADT. We identified a set of nearly 700 genes which were reciprocally regulated between the two datasets and so were strong candidates to be clinically relevant androgen-regulated genes in prostate cancer. This set of 700 genes included the gene for the cancer-associated cell migration protein Tetraspanin 1 (TSPAN1) which had not previously been shown to be regulated by androgens in prostate cancer.

Tetraspanins, also known as the transmembrane 4 superfamily, are small transmembrane glycoproteins which were first described in studies of tumour associated proteins^8–13^. As a member of the tetraspanin family, TSPAN1 has been reported to regulate cancer progression in many human cancers. TSPAN1 is upregulated in human hepatocellular carcinoma^14^, gastric carcinoma^15^, colorectal adenocarcinoma^16^, ovarian carcinomas^17^ and cervical cancer^18, 19^. Tetraspanins reportedly play a role in a range of biological processes including cell proliferation^9^, cell adhesion^20^, cell migration and motility^21, 22^ and signal transduction^23, 24^. Here, we show that expression of TSPAN1 is controlled by androgens in prostate cancer cells, is upregulated in prostate cancer tissue and is important for prostate cancer cell survival and migration. Our findings are in agreement with numerous studies showing that TSPAN1 is upregulated in several other cancer types^15, 17, 25–28^, but are in contrast to a recent publication suggesting that decreased TSPAN1 is linked to prostate cancer progression^29^.

## Results

### TSPAN1 is an early target of the AR *in vitro* and *in vivo*

**Figure 1:**
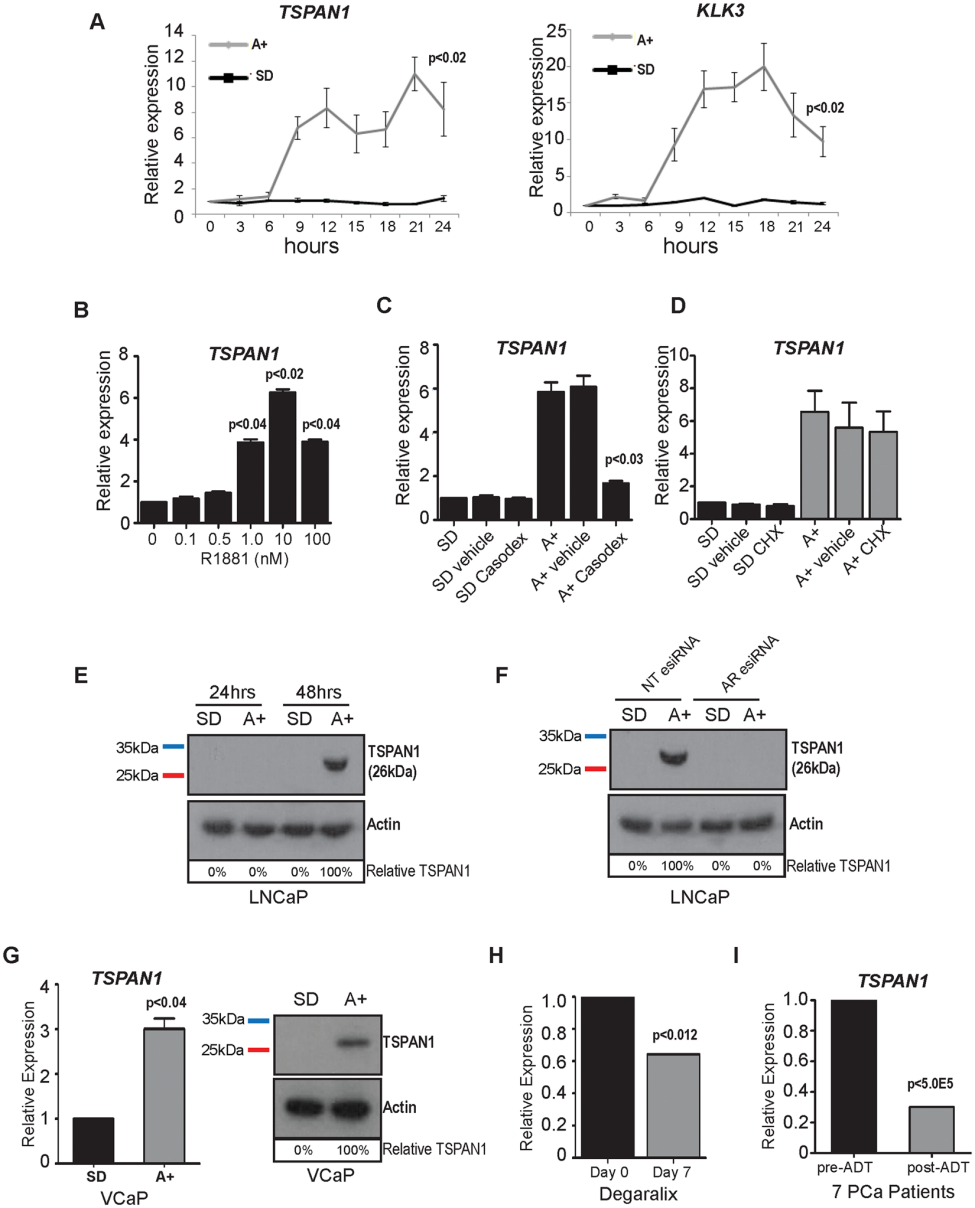
TSPAN1 is regulated by androgens in *in vitro* and *in vivo*. (**A**) Analysis of *TSPAN1* and *KLK3* (PSA) mRNA in LNCaP cells treated with androgens (A+) over a 24 hour time course. (**B**) Upregulation of *TSPAN1* is also evident in LNCaP cells treated with 1 to 100 nM of R1881. (**C**) Induction of TSPAN1 mRNA by the AR is inhibited in the presence of 10 μM of the anti-androgen Casodex (bicalutamide) (lane 6). (**D**) The increase in *TSPAN1* mRNA expression in response to androgens is still seen in the presence of 1 μg/ml cycloheximide (CHX). Relative *TSPAN1* expression was detected by real-time PCR and normalised to three reference genes. (**E**) Expression of TSPAN1 protein is induced in LNCaP cells treated with 10 nM R1881 for 48 hours as detected by western blot. Actin was used as a loading control. (**F**) Depletion of AR protein in LNCaP cells by esiRNA shows that when the AR is depleted there is no induction of the TSPAN1 protein in response to androgens. (**G**) Real-time PCR and western blot analysis of *TSPAN1* mRNA and protein in VCaP cells grown in steroid deplete (SD) or 10 nM R1881 (androgens, A+) treated conditions for 48 hours. (**H**) Analysis of *TSPAN1* mRNA expression before and after 7 days of serum/tissue androgen depletion in prostate cancer patients using degarelix^31^. (**I**) Analysis of *TSPAN1* expression in previously published RNA-Seq data^32^ shows that TSPAN1 mRNA is significantly down-regulated in 7 PCa patients following androgen deprivation treatment (ADT). The Y axis shows the relative expression level of TSPAN1 (calculated by comparing the FPKM expression values of genes after ADT to the value before treatment). Please note: cropped western blots are displayed in the figure, but full length blots are included in the Supplementary Information file.

Previous RNA-Seq analysis of LNCaP cells identified the cancer-associated *TSPAN1* gene as being under control of androgens after 24 hours treatment with 10 nM of the synthetic androgen analogue R1881 (methyltrienolone)^7^. Using a time course and real-time PCR we found that androgen mediated induction of the *TSPAN1* gene could be detected in LNCaP cells 9 hours after androgen exposure suggesting it is directly regulated by the AR. The early expression profile of *TSPAN1* following androgen exposure had similar dynamics to the known directly AR-regulated gene *KLK3 (PSA)* (Fig. 1A). Androgen-mediated induction of *TSPAN1* expression in LNCaP cells was also induced by treatment with a range of R1881 concentrations for 24 hours, consistent with *TSPAN1* induction also occurring under physiological androgen concentrations within the prostate (Fig. 1B), and was blocked by treatment with the AR antagonist Casodex^R^ (bicalutamide) (Fig. 1C). To test whether androgen-mediated induction of TSPAN1 expression was a result of AR activity, we treated LNCaP cells with 10 mM R1881 (androgens) in the presence and absence of cycloheximide to inhibit de novo protein synthesis. Androgen mediated up-regulation of TSPAN1 mRNA expression was observed in the presence or absence of the protein synthesis inhibitor cycloheximide indicating that TSPAN1 induction might be directly mediated by the AR (Fig. 1D). Consistent with this, analysis of previously published AR ChIP-Seq data^30^ revealed an AR binding site which is overlapping with the start of the TSPAN1 gene for both LNCaP and VCaP cells (Supplementary Figure 1).

Expression of TSPAN1 protein was also induced by treatment of LNCaP cells with 10 nM R1881 (Fig. 1E). Confirming this effect on protein levels was mediated by the AR, we found that androgen-mediated induction of the TSPAN1 protein is prevented when cells are depleted of the AR using esiRNA (Fig. 1F). Interestingly, although induction of TSPAN1 mRNA was detected within 9 hours of androgen exposure, induction at the protein level was not detected until 48 hours. Specificity of our TSPAN1 antibody was confirmed via siRNA mediated protein depletion and detection of over-expressed protein by western blotting (Supplementary Figure 2A,B). Supporting our data obtained in LNCaP cells, TSPAN1 expression was also androgen responsive in VCaP cells at both the RNA and protein level (Fig. 1G). Analysis of previously published RNA-Seq data from before and after 7 days of androgen depletion in prostate cancer patients using Degarelix^31^ shows a significant reduction in *TSPAN1* mRNA indicating a tissue based response (Fig. 1H). Similarly, analysis of previously published RNA-Seq data from 7 prostate cancer patients^32^ showed that *TSPAN1* gene expression is strongly down-regulated following ADT (Fig. 1I), suggesting that *TSPAN1* is also regulated (either directly or indirectly) by androgens *in vivo* within patients.

### TSPAN1 is upregulated in prostate cancer tissue

**Figure 2:**
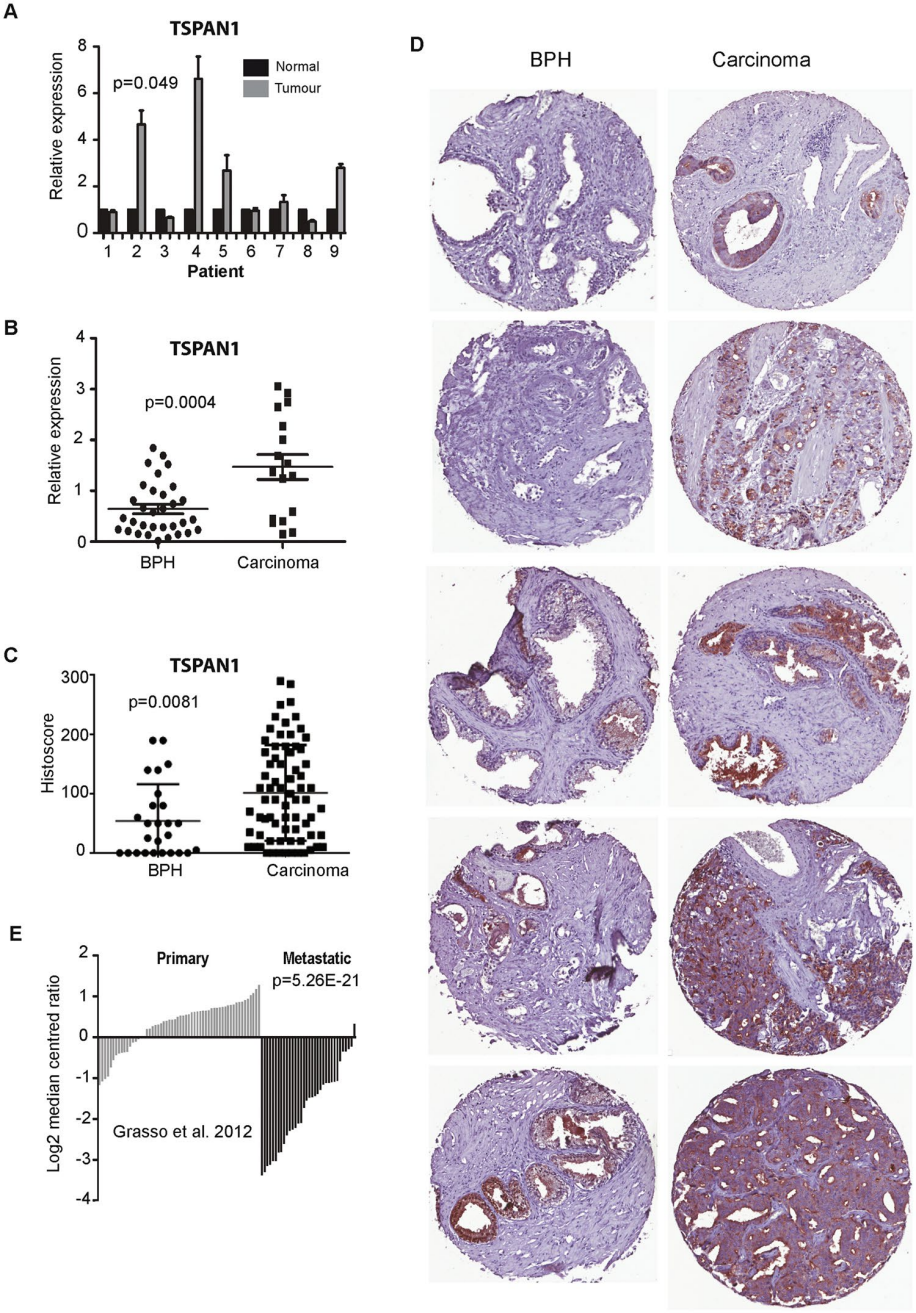
TSPAN1 is upregulated in prostate cancer tissue. (**A**) Real-time PCR analysis of *TSPAN1* mRNA from normal and matched prostate cancer tissue from 9 patients obtained from radical prostatectomy. **(B)** We also analysed 32 benign samples from patients with benign prostate hyperplasia (BPH) and 17 malignant samples from transurothelial resection of the prostate (TURP) samples. **(C)** Analysis of TSPAN1 protein levels in patients with BPH or prostate cancer by Tissue Microarray (TMA). **(D)** Representative cores from each TMA. **(E)** *TSPAN1* gene expression levels in prostate tissue from the primary or metastatic site in data published by Grasso *et al*.^33^.

We carried out meta-analysis of 1012 prostate tissue samples using data from 14 previously published studies^33–46^ to monitor how expression of *TSPAN1* changes in clinical prostate cancer. We found that 11/14 datasets showed significant up-regulation of *TSPAN1* mRNA expression in prostate carcinoma versus normal prostate tissue (Supplementary Table 1). *TSPAN1* expression showed an average mean fold change of 2.214 (p = 1.31E-6) in 122 primary prostate carcinoma samples studied by Grasso *et al*.^33^, and a mean fold change of 1.844 (p = 1.72E-7) in 185 samples studied by Taylor *et al*.^34^. *TSPAN1* expression analysis by real-time PCR showed that *TSPAN1* mRNA was significantly up-regulated in prostate carcinoma samples relative to BPH samples (p = 0.049), and in primary prostate tumour tissue relative to matched normal tissue from the same patient (p = 0.0004) (Fig. 2A,B).

To develop a rigorous test for TSPAN1 protein we extensively validated our TSPAN1 antibody in multiple cell lines using western blotting, siRNA mediated protein depletion, and detection of over-expressed protein (Supplementary Figure 2A,B). To examine TSPAN1 protein expression in clinical prostate cancer tissue we further showed that staining by this antibody was blocked by pre-incubation with a blocking peptide (Supplementary Figure 2C) and by detection of siRNA mediated protein depletion by immunohistochemistry in Formalin Fixed Paraffin Embedded (FFPE) cell pellets (Supplementary Figure 2D). TSPAN1 is predicted to be a transmembrane protein (http://www.uniprot.org/uniprot/O60635#subcellular_location), and the localisation of TSPAN1 within prostate tissue was mainly cytoplasmic and membrane bound. Staining a tissue microarray (TMA) containing 26 samples from patients with BPH and 72 samples from patients with prostate cancer confirmed that TSPAN1 protein is also expressed at significantly higher levels in prostate cancer patients compared to benign controls (p = 0.0081) (Fig. 2C) (further information on the clinical samples used in the TMA is given in Supplementary Table 2). Representative cores from each TMA are shown in Fig. 2D.

A previous study suggested decreased TSPAN1 could be used to predict biochemical recurrence after radical prostatectomy^29^, however, our data indicates no significant correlation between both overall survival and relapse free survival for patients with either low or high TSPAN1 (Supplementary Figure 3). To determine whether TSPAN1 is associated with cancer metastasis, we studied *TSPAN1* gene expression in prostate cancer tissue obtained from either the primary or metastatic site using a previously-published clinical dataset^33^. We observed a changing pattern of TSPAN1 expression between primary and metastatic tumours with a striking decrease in TSPAN1 expression in prostate cancer tissue from the metastatic site (p = 5.26E-21) (Fig. 2E).

### TSPAN1 increases prostate cancer cell migration and can upregulate expression of Slug and ARF6

**Figure 3:**
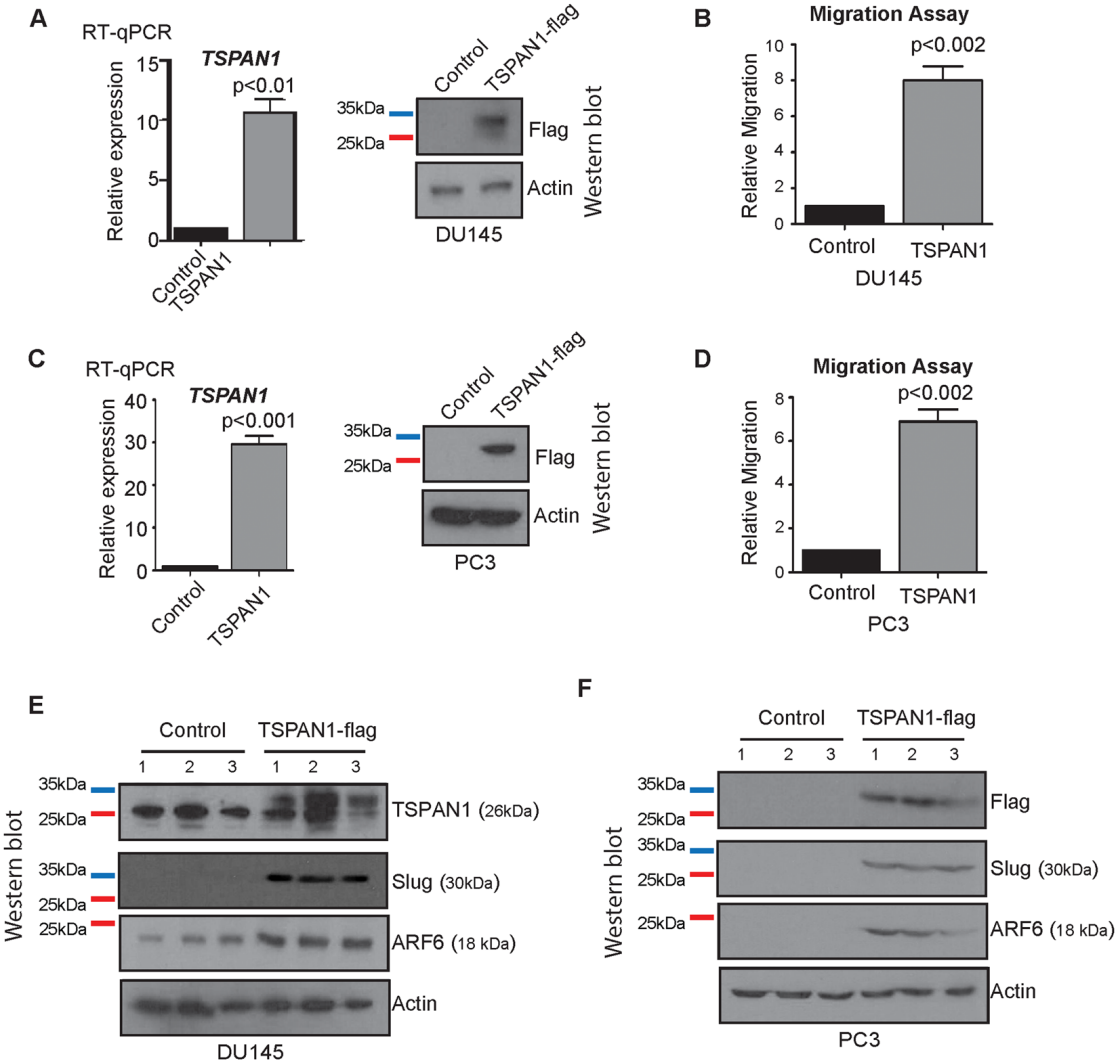
TSPAN1 increases prostate cancer cell migration and induces the expression of Slug and ARF6. (**A,C**) Validation of TSPAN1 overexpression in DU145 and PC3 cells by real-time PCR and western blot. **(B,D)** Migration assays for DU145 and PC3 cells overexpressing TSPAN1. **(E,F)** Western blot analysis of Slug and ARF6 protein expression in DU145 and PC3 cells overexpressing TSPAN1. Please note: cropped western blots are displayed in the figure, but full length blots are included in the Supplementary Information file.

The above data indicated that prostate cancer cells over-express TSPAN1 compared to normal or benign prostate tissue. To investigate why upregulation of TSPAN1 might be important in prostate cancer cells, we created DU145 and PC3 cell lines that over-express TSPAN1 protein (Fig. 3A,C). DU145 and PC3 cells were chosen for these analyses as they have low endogenous TSPAN1 levels (Supplementary Figure 4). Although there were no significant differences in cell proliferation or adhesion with upregulated TSPAN1 (Supplementary Figure 5), the rate of cell migration was significantly increased in both DU145 and PC3 cell backgrounds (Fig. 3B,D).

To probe the regulatory mechanism governing TSPAN1-dependent prostate cancer cell motility, we analysed expression of 77 genes with established roles in the epithelial to mesenchymal transition (EMT) and/or cell motility and migration. In DU145 cells 10 out of 77 genes showed significant changes in gene expression when TSPAN1 was overexpressed (Supplementary Table 3), including genes associated with EMT. Increased expression of the EMT-related transcriptional repressor Slug and the small GTP-binding protein ARF6 was also seen at the protein level (Fig. 3E), and in PC3 cells with upregulated TSPAN1 (Fig. 3F). Slug expression is increased in advanced-stage primary prostate cancer where it has been shown to play a role in the EMT transition^47^. Slug is also highly expressed in prostate cancers associated with a neuroendocrine phenotype^48^. Consistent with this, using data generated by Beltran *et al*.^49^ we found TSPAN1 and Slug are co-amplified in 28% and 37% of neuroendocrine prostate cancer tumours respectively (Supplementary Table 4).

### TSPAN1 is essential for prostate cancer cell viability

**Figure 4:**
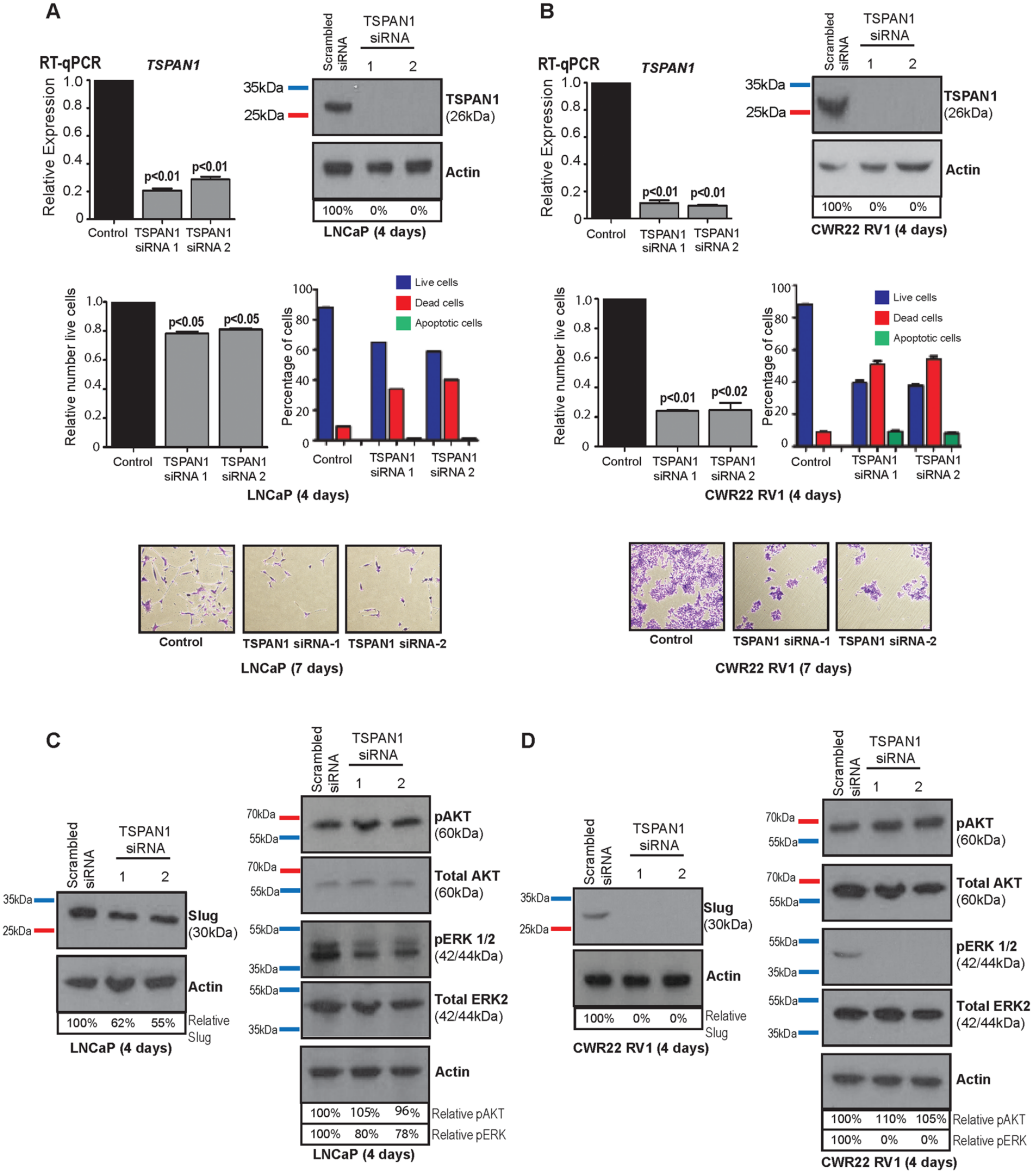
TSPAN1 is essential for prostate cancer cell viability. Depletion of TSPAN1 in **(A)** LNCaP and **(B)** CWR22 RV1 cells by transient transfection with siRNA. Protein depletion was carried out using two different siRNAs in cells grown in full media. 96 hours after transfection knockdown of TSPAN1 was confirmed by real-time PCR and western blot and the relative number of live cells was calculated. Representative crystal violet stained images are shown below for each cell line after 7 days (due to a dramatic decrease in cell viability we were unable to harvest enough live cells to confirm TSPAN1 knockdown after 96 hours). **(C)** Western blot analysis of various proteins in LNCaP and **(D)** CWR22 RV1 cells following siRNA mediated protein depletion of TSPAN1 for 96 hours. Please note: cropped western blots are displayed in the figure, but full length blots are included in the Supplementary Information file.

Previous studies on other cancer cell types have indicated that TSPAN1 can be essential for cell survival and proliferation^28, 50, 51^. To test whether changes in the expression of TSPAN1 can influence prostate cancer cell viability, we depleted LNCaP and CWR22RV1 cells of TSPAN1 protein using two independent siRNAs. Loss of TSPAN1 resulted in a significant reduction in cell number and significantly reduced cell viability for both cell lines 96 hours after siRNA treatment (Fig. 4A,B). To examine whether TSPAN1 is upstream of key cell signalling pathways in prostate cancer, we tested whether depletion of TSPAN1 influenced PIK3 signalling (detected using phospho-AKT) or RAS/ERK1/2 signalling (detected using phospho-ERK1/2). We observed a significant reduction in the levels of phospho-ERK1/2 levels in both LNCaP and CWR22RV1 cells in LNCaP cells with depleted TSPAN1, but there was no difference in the levels of phospho-AKT (Fig. 4C,D).

## Discussion

The progression to advanced prostate cancer is thought to involve persistence of AR signalling, and prostate cancer treatment is dominated by strategies to control AR activity^52^. The AR controls prostate cancer development through the regulation of transcription, meaning that identifying both transcriptional targets of the AR and the factors involved will provide opportunities for both cancer detection and therapeutic intervention. Here, we show that the Tetraspanin 1 gene (*TSPAN1*) is regulated by androgens and is significantly upregulated in primary prostate cancer tissue. We find that while normal expression of TSPAN1 is important for prostate cancer cell viability, induced upregulated expression drives increased cell migration. The data presented in this study are in contrast to a recent publication suggesting that decreased TSPAN1 is linked to prostate cancer progression and that TSPAN1 plays a negative role in prostate cancer cell proliferation and migration (which used a different antibody to detect TSPAN1^29^), but are in agreement with studies in numerous other cancer types where upregulated TSPAN1 is linked to cancer progression, cell survival, proliferation, migration and invasion^15, 17, 25–28, 50, 51, 53^.

Tetraspanins are reported to play a role in a range of biological processes including cell proliferation^9^, cell adhesion^20^, cell migration and motility^11, 21, 22^ and signal transduction^23, 24^. Previous studies have indicated that tetraspanins can regulate the function of proteins involved in all aspects of cell migration^11^. For example, tetraspanins can directly interact with integrins and modulate their downstream signalling in response to migratory signals^54, 55^. Tetraspanin CD9 has been shown to regulate expression of MMP9 via the JNK pathway^56, 57^, and tetraspanin CD82 can inhibit cancer cell retraction and motility via deregulation of the Rac1/RhoA signaling network^58, 59^. Tetraspanins also play a role in E-cadherin based cell–cell junctions^60–62^, and can directly interact with and influence the activity of matrix metalloproteinases^63, 64^.

TSPAN1 has been shown to promote invasiveness of cervical cancer cells^27^, increase the proliferation and invasion of colon cancer cells *in vitro* ^50^, promote survival and invasion of skin carcinoma cells^51^, and to play a role in survival, proliferation and carcinogenesis of pancreatic cancer^28^. In addition, regulation of TSPAN1 by micro-RNAs has been shown to promote cell proliferation and invasion in colorectal cancer^65^, and cell migration in non-small cell lung cancer^26^. Taken together, these studies suggest a positive role for TSPAN1 in cancer progression, but the mechanisms underlying this are currently poorly understood. Here, we find for the first time, that TSPAN1 expression controls the expression of key proteins with roles in cell migration. We show that upregulation of TSPAN1 in prostate cancer cells results in increased expression of the zinc finger transcription factor Slug (SNAI2) which has previously been shown to promote migration and invasion, and a transition to a more mesenchymal state in prostate cancer cells^47, 66^. Slug expression is increased in advanced primary prostate tumours^47^, and clusters in cancer cells at the invasive front in neuroendocrine areas of high-grade prostate cancer^48^. TSPAN1 also modulates the expression of small GTPase ADP-ribosylation factor 6 (ARF6) which is linked to cancer cell motility^67^, and is upregulated in prostate cancer tissue^68^. To the best of our knowledge this is the first time these genes have been shown to be regulated by TSPAN1 in any cancer type. Inhibition of TSPAN1 in prostate cancer has been previously linked with increased phosphorylation of AKT^29^, however we found no evidence of this in our study. A study in cervical cancer excluded a role for TSPAN1 in focal adhesion kinase (FAK), phosphoinositide-3-kinase (PI3K) and in EGFR-dependent signalling to the RAS/ERK pathway, arguing against a role for TSPAN1 as a mediator of cell surface receptor signalling^27^.

By identifying a mechanism through which this protein can modulate cell migration this current study thus sheds new light on the role of TSPAN1 in cancer. EMT and MET are processes by which cells transit between epithelial and mesenchymal states and play an integral role in cancer metastasis^69^. Activation of EMT is important for cancer cell dissemination, whereas the reversal of EMT (or MET) is necessary for efficient metastatic colonisation^70, 71^. Taken together our data suggest dynamic TSPAN1 expression levels might contribute to transitions between a more migratory mesenchymal like cell phenotype and establishing stable metastases. We find TSPAN1 expression in prostate cancer is biphasic, with upregulation at the primary site and downregulation in metastatic lesions. Similarly, a study in breast cancer has shown that although TSPAN1 is upregulated in most primary tumours it is more likely to be downregulated in metastatic lesions^72^.

TSPAN1 is part of a larger gene family. It has been speculated that distinct combinations of tetraspanins could act in concert to control various aspects of cell behaviour in cancer cells^27^. Tetraspanin CD151 is elevated in patients with prostate cancer^73^ and has been shown to contribute to spontaneous metastasis in mouse models^74^. TSPAN8 is also upregulated in prostate cancer tissue and associated with cell invasion^75^. Conversely, tetraspanin CD9 is downregulated during prostate cancer progression^76^ and is a suppressor of metastasis in prostate cancer mouse models^77^. Prostate cancer is an extremely heterogeneous disease and it is likely that the role of tetraspanins is multifaceted in the development and progression of prostate cancer. Interestingly, TSPAN1 is listed in the top 30 most common and abundant glycoproteins detected in prostate cancer secretions by mass spectrometry and has been studied as part of a predictive signature for prostate cancer aggressiveness^78, 79^. The overlapping TMA data for TSPAN1 between benign and prostate cancer groups presented in this study do not suggest that detection of TSPAN1 alone could be used clinically, but indicate it may still be worthwhile to examine TSPAN1 levels as part of a ‘tetraspanin signature’ in future studies.

In conclusion, our study shows that expression of TSPAN1 is controlled by androgens in prostate cancer cells and is upregulated in prostate cancer tissue. The AR has been shown to be essential for prostate cancer cell viability, proliferation and invasion^80–82^, however the mechanisms controlling this are poorly understood. Our data supports TSPAN1 as an androgen-driven contributor to prostate cancer cell survival and motility, which can control the expression of the mesenchymal proteins Slug and ARF6. Future studies will help elucidate the role TSPAN1 plays in these processes in relation to other AR regulated genes and whether TSPAN1 can be targeted therapeutically.

## Methods

### Cell Culture

Cell culture and the treatment of cells with androgens were as described previously^7, 83–86^. LNCaP cells (CRL-1740, ATCC) were maintained in phenol red free RPMI-1640 with L-Glutamine (PAA Laboratories, R15–802) supplemented with 10% Fetal Bovine Serum (FBS) (PAA Laboratories, A15-101). For androgen treatment of LNCaP cells, medium was supplemented with 10% dextran charcoal stripped FBS (PAA Laboratories, A15-119) to produce a steroid-deplete medium. Following culture for 72 hours, 10 nM synthetic androgen analogue methyltrienolone (R1881) (Perkin–Elmer, NLP005005MG) was added (Androgen+) or absent (Steroid deplete) for the times indicated. Stable cell lines were generated by transfecting cells using Lipofectamine 2000 (11668-027, Invitrogen), followed by selection with 300 µg/ml Geneticin (Invitrogen, 10131019) (reduced to 150 µg/ml following the death of untransfected cells) for at least four weeks.

### siRNA

Knockdown of TSPAN1 was carried out using predesigned siRNA sequences purchased from IDT (hs.Ri.TSPAN1.13.1 and hs.Ri.TSPAN1.13.2). AR esiRNA was obtained from Sigma-Aldrich (EHU025951). Transfections were carried out using Lipofectamine^R^ RNAi Max (Thermo Fisher Scientific 13778075) as per the manufacturer’s instructions.

### RT-qPCR

Cells were harvested and total RNA extracted using TRI-reagent (Invitrogen, 15596-026), according to the manufacturer’s instructions. RNA was treated with DNase 1 (Ambion) and cDNA was generated by reverse transcription of 200ng of total RNA using the Superscript VILO cDNA synthesis kit (Invitrogen, 11754-050). Quantitative PCR (qPCR) was performed in triplicate on cDNA using SYBR® Green PCR Master Mix (Invitrogen, 4309155) using the QuantStudio™ 7 Flex Real-Time PCR System (Life Technologies). Gene expression changes were analysed using relative quantification (comparative CT method) to a given control sample. Samples were normalised using the average of three reference genes: GAPDH, β –tubulin and actin. All primer sequences are listed in Supplementary Table 5.

### Antibodies

The following antibodies were used in the study: anti-TSPAN1 antibody produced in rabbit (Sigma-Aldrich HPA011909), anti-AR mouse antibody (BD Bioscience, 554226), anti-p21 Waf1/Cip1 rabbit antibody (Cell Signaling 2947), anti-slug rabbit antibody (Cell Signaling 9585), anti- phospho-p44/42 MAPK mouse monoclonal (Erk1/2 Thr202/Tyr204) (Cell signaling 9106), anti-ERK2 mouse monoclonal (1647 Santa Cruz), anti-AKT rabbit antibody (Santa Cruz, sc-8312), anti-phospho-AKT1 (pSer473) rabbit antibody (Sigma, SAB4300042), anti-ARF6 rabbit antibody (Cell Signaling 5740), anti-FLAG mouse monoclonal (F3165, Sigma), anti-actin rabbit polyclonal (A2668, Sigma), normal rabbit IgG (711-035-152 Jackson labs) and normal mouse IgG (715-036-150 Jackson labs). Quantifications of western blots were carried out using ImageJ. Pixel densities for the bands of interest were calculated relative to background levels and the normalising control (actin). The concentrations of antibodies used are given in Supplementary Table 6.

### DNA Constructs

For stable transfection of DU145 and PC3 cells TSPAN1 was cloned into pX3flag CMV 10 (E7658, Sigma) using EcoR1 and Xba1. The plasmid used underwent DNA sequencing to confirm correct insertion of the TSPAN1 gene.

### Cell Viability Assay

Cell viability analysis was carried out using the TaliR Cell Viability Kit (Life Technologies A10786) and the TaliR Image-based Cytometer. Relative cell numbers following siRNA treatment were determined using the TaliR Image-based Cytometer (Life Technologies).

### Migration Assays

Cell migration assays were carried out using the collagen coated Oris^TM^ Pro Cell Migration Assay (Platypus Technologies PROCMACC1) as per the manufacturer’s instructions. For both DU145 and PC3 cells 30,000 cells per well were seeded at the start of the experiment. The results presented show the relative migration for each cell line over 41 hours (DU145 cells) or 30 hours (PC3 cells).

### Adhesion Assays

For adhesion assays cells were labelled with 5 µM Calcein-AM (BD Biosciences). 50,000 cells were then allowed to adhere to collagen coated plates for two hours, washed 4 times with PBS (to remove any non-adherent cells), and the absorbance measured at 485/538 nm using a plate reader. The percentage adhesion was then calculated by comparing a washed plate (representative of the number of adherent cells) to an identical unwashed plate (representative of the total number of cells seeded).

### Immunohistochemistry

A tissue microarray (TMA) containing 0.6 mm cores of benign prostatic hyperplasia (BPH) (n = 26), PC (n = 72), and control tissues including breast, kidney, placenta, ovary, and liver was used^87^. A specialised urological pathologist selected representative tissue pieces to be included in the TMA based on percentage of tumours cells assessed from the H&E stained sections. For each patient two separate representative areas were selected and included on the TMA to account for tumour heterogeneity. Antigen retrieval was performed by pressure cooking the TMA for 90 seconds in 10 mM citrate pH 6.0 followed by staining the tissues with anti-TSPAN1 antibody (Sigma-Aldrich HPA011909) at a 1:1500 dilution. Nuclei were counterstained with haematoxylin. The TMA was scored by Urszula L. McClurg using the 0-300 Histoscore score method^88^. Only epithelial cells were scored. Sections were scored based on their staining intensity with 0 being assigned to cells with absent staining, 1 to weak staining, 2 to moderate staining and 3 to strong staining. Within each staining intensity percentage of epithelial cells (0–100%) with this staining intensity was assigned. This resulted in a Histoscore calculated from the following equation H = 0x (% of cells scored at 0) + 1x (% of cells scored at 1) + 2x (% of cells scored at 2) + 3x (% of cells scored at 3).

### Clinical Samples

Our study made use of RNA from 32 benign samples from patients with benign prostatic hyperplasia (BPH) and 17 malignant samples from transurethral resection of the prostate (TURP) samples. Malignant status and Gleason score were obtained for these patients by histological analysis. We also analysed normal and matched prostate tissue from 9 patients obtained by radical prostatectomy. The samples were obtained with ethical approval through the Exeter NIHR Clinical Research Facility tissue bank (Ref: STB20). Our TMA study made use of 26 BPH and 72 prostate cancer tissue samples. These samples were obtained with full ethical approval from the Northumberland, Tyne and Wear NHS Strategic Health Authority Local Research Ethics Committee (Ref: 2003/11). Written informed consent for the use of surgically obtained tissue was provided by all patients. All methods were performed in accordance with the relevant guidelines and regulations.

## Electronic supplementary material

Supplementary Information
