# Supplementary material for: The cancer-associated cell migration protein TSPAN1 is under control of androgens and its upregulation increases prostate cancer cell migration
Source: Sci Rep. 2017 Jul 12;7:5249. doi: 10.1038/s41598-017-05489-5 (PMC5507901; doi:10.1038/s41598-017-05489-5)
Supplement: Supplementary file 1 — Supplementary Information [file 41598_2017_5489_MOESM1_ESM.pdf]

The cancer-associated cell migration protein TSPAN1 is under direct control of androgens and its upregulation increases prostate cancer cell migration

Jennifer Munkley[1]*, Urszula L. McClurg[2], Karen E. Livermore[1], Ingrid Ehrmann[1], Bridget Knight[3], Paul Mccullagh[4], John Mcgrath[5], Malcolm Crundwell[6], Lorna W. Harries[7], Hing Y. Leung[8,9], Ian G. Mills[10,11,12], Craig N. Robson[2], Prabhakar Rajan[13] & David J. Elliott[1]

Supplementary Information:

Supplementary Data

Supplementary Figure 1: Androgen Receptor ChIP-Seq peaks in LNCaP and VCaP cells identified by Massie et al. 2011.

Supplementary Figure 2: TSPAN1 antibody validation.

Supplementary Figure 3: (A) Overall survival and relapse free survival for patients with either low or high TSPAN1. (B) TSPAN1 histoscores correlation with prostate cancer patient clinical parameters: i) PSA levels at patient relapse, ii) hormone sensitive or castrate resistant disease.

Supplementary Figure 4: Relative endogenous TSPAN1 levels in prostate cancer cell lines

Supplementary Figure 5: Analysis of cell proliferation and cell adhesion in DU145 cells stably transfected with TSPAN1.

Supplementary Table 1: Meta-analysis of TSPAN1 expression in prostate cancer tissue.

Supplementary Table 2: Basic clinical data for patient tissue samples used in TMA

Supplementary Table 3: Real-time PCR analysis of 77 cell motility genes in DU145 cells stably transfected with TSPAN1.

Supplementary Table 4: Differential analysis of gene expression in neuroendocrine prostate cancer (data produced by Beltran et al. 2016 44).

Supplementary Table 5: Real-time PCR primer sequences used.

Supplementary Table 6: Concentrations of antibodies used.

Full length western blots are also included for supplementary information

Supplementary Figure 1

Androgen Receptor ChIP-Seq peaks in LNCaP and VCaP cells identified by Massie et al. 2011

LNCaP cells

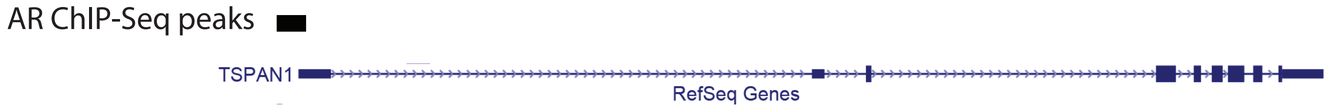

TSPAN1 chr1:46640749-46651634

VCaP cells

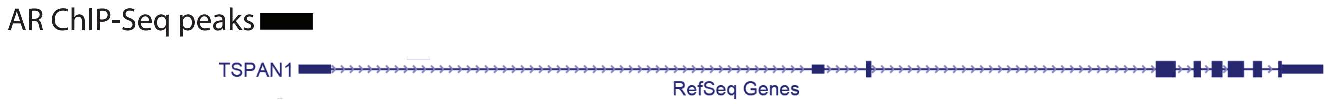

TSPAN1 chr1:46640749-46651634

Predicted AR binding sites:

LNCaP cells: chr1: 46640495-46640802

VCaP cells: chr1:46640361-46640945

Supplementary Figure 2

TSPAN1 antibody validation

A

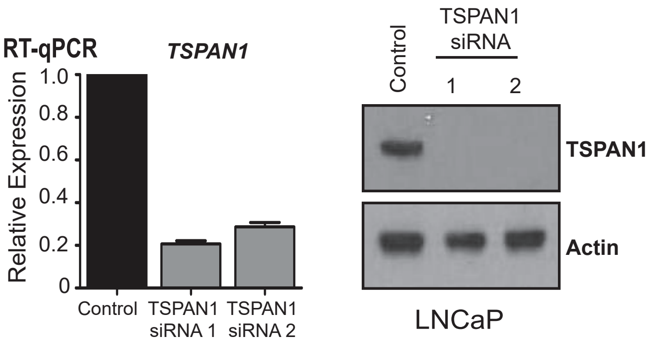

(duplicated from Figure 3A)

B

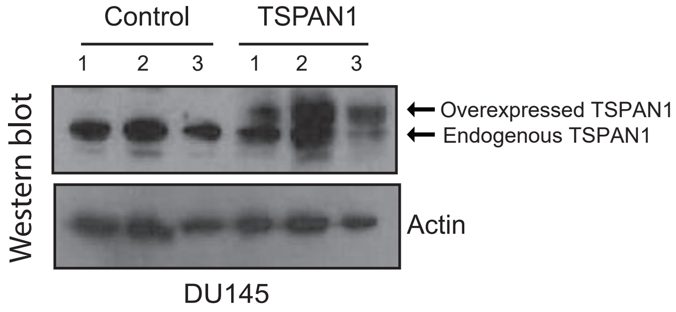

C

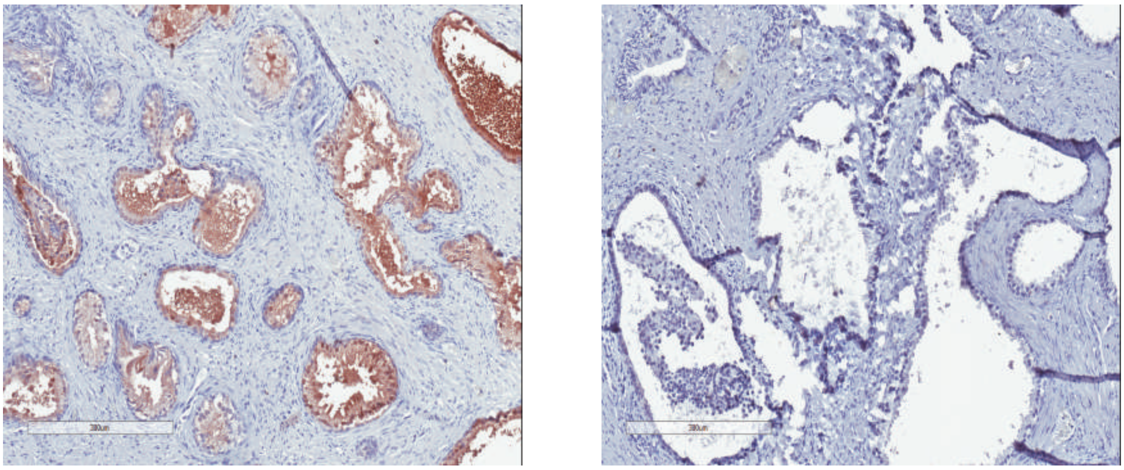

Prostate cancer tissue stained with TSPAN1 antibody at 1:1500

Prostate cancer tissue stained with TSPAN1 antibody at 1:1500 following pre-incubation with TSPAN1 immunising peptide

D

Control siRNA

TSPAN1 siRNA

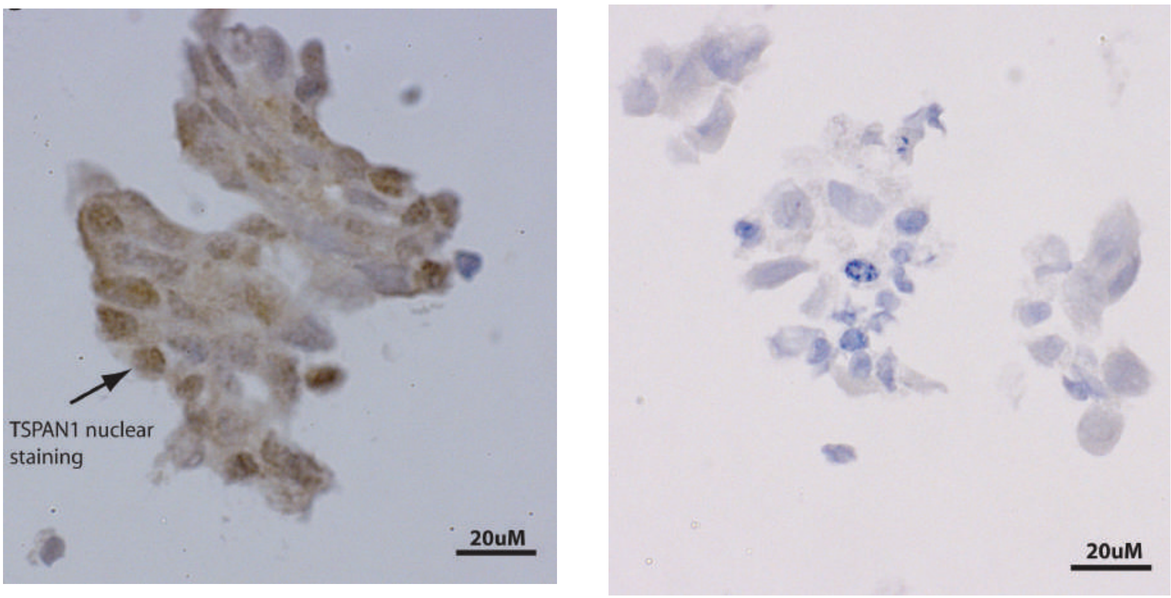

Detection of siRNA mediated depletion of TSPAN1 in FFEP LNCaP cell pellets stained with TSPAN1 antibody

Supplementary Figure 3A

Overall survival and relapse free survival for patients with either low or high TSPAN1

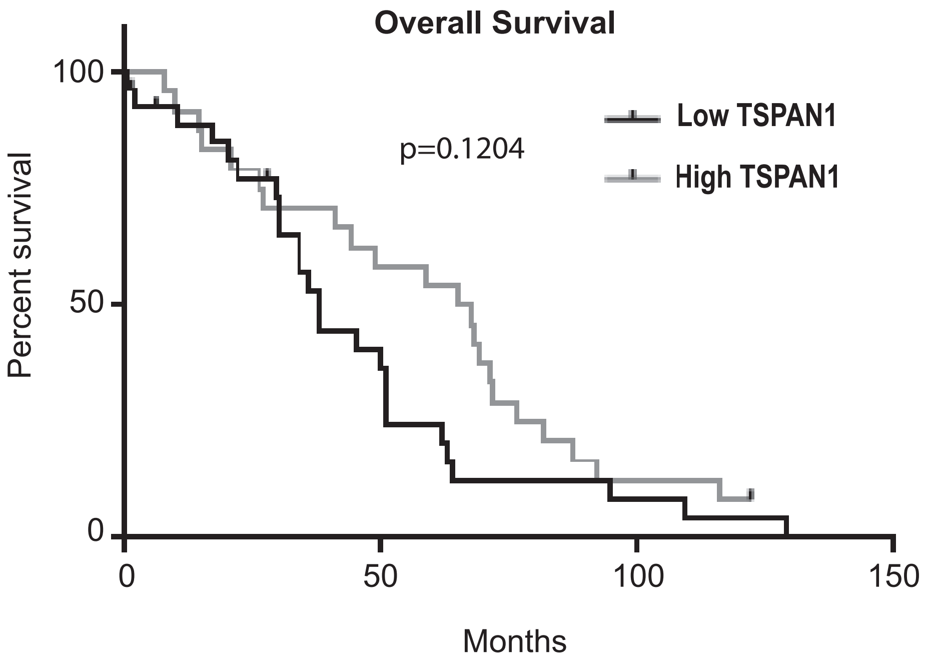

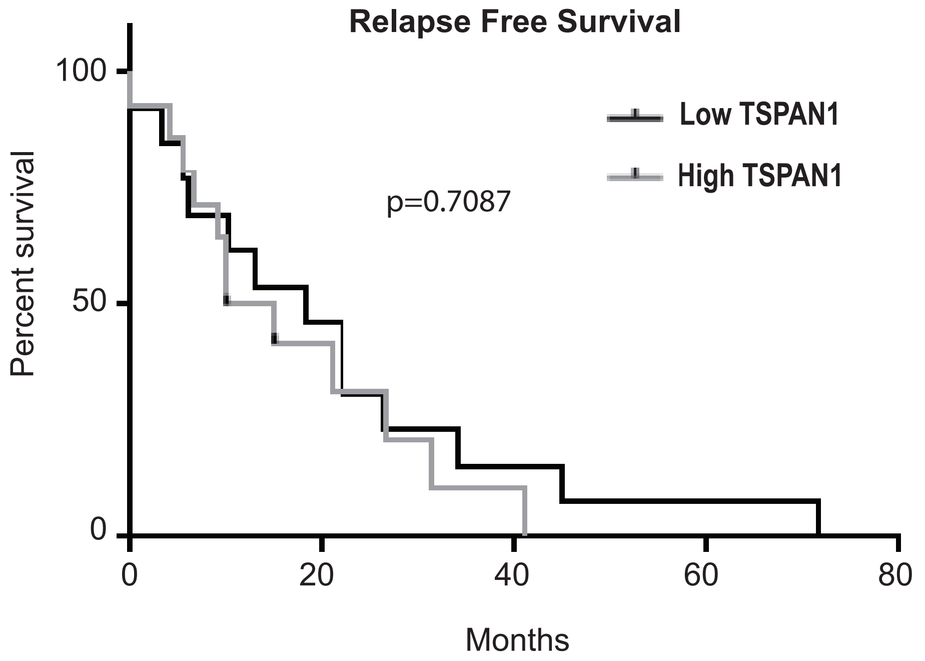

Supplementary Figure 3B

TSPAN1 histoscores correlation with prostate cancer patient clinical parameters

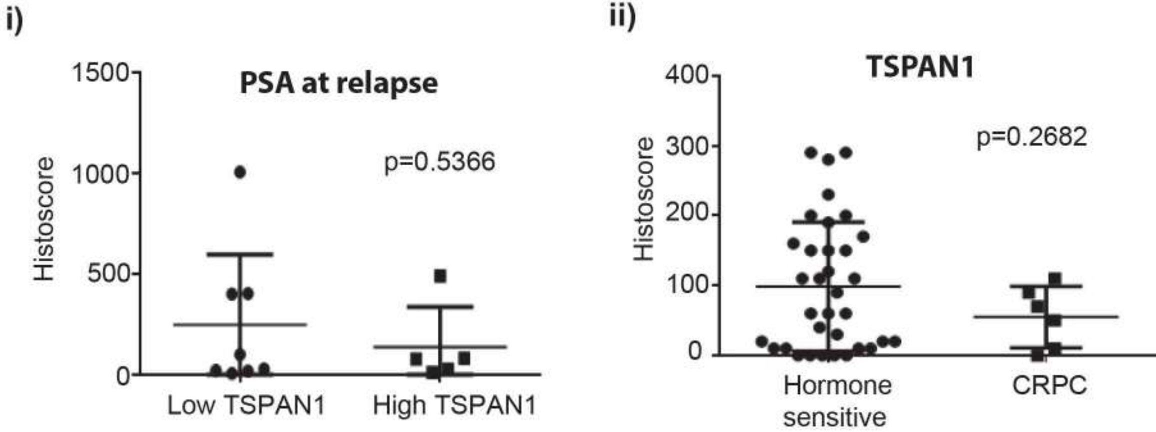

Supplementary Figure 4

Relative TSPAN1 expression levels in different prostate cancer cell lines

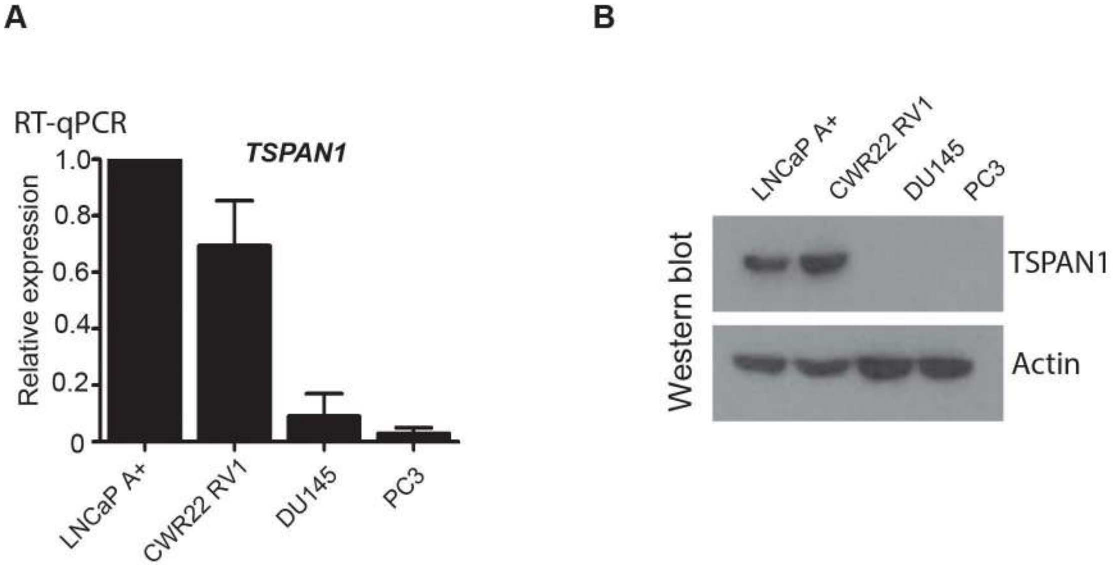

Supplementary Figure 5

Analysis of cell proliferation and cell adhesion in DU145 cells stably transfected with TSPAN1

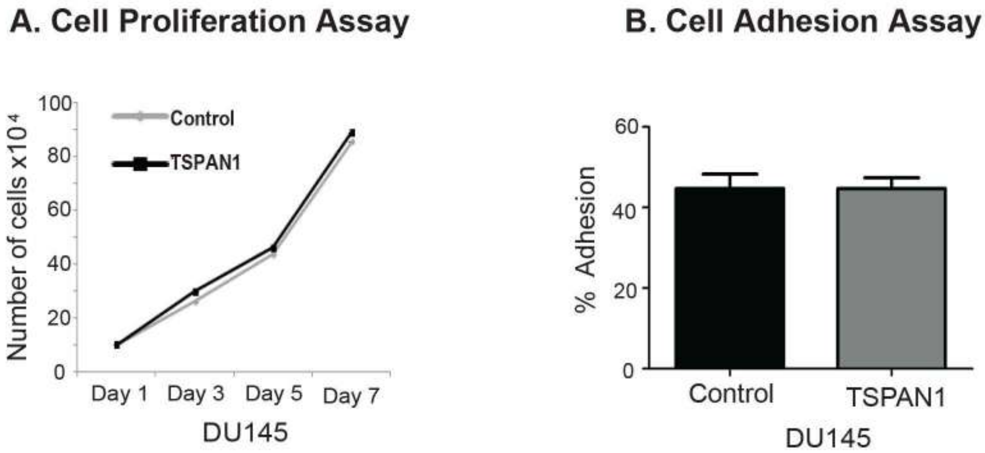

Munkley et al. Full western blot images

The cancer-associated cell migration protein TSPAN1 is under direct control of androgens and its upregulation increases prostate cancer cell migration

Figure 1D

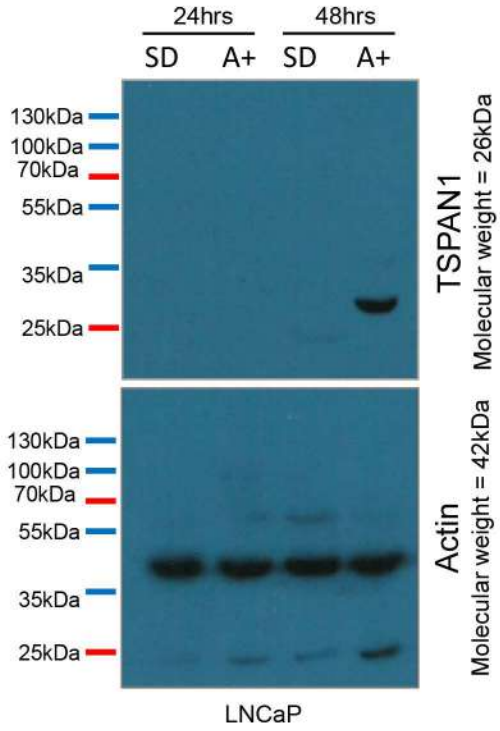

Figure 1E

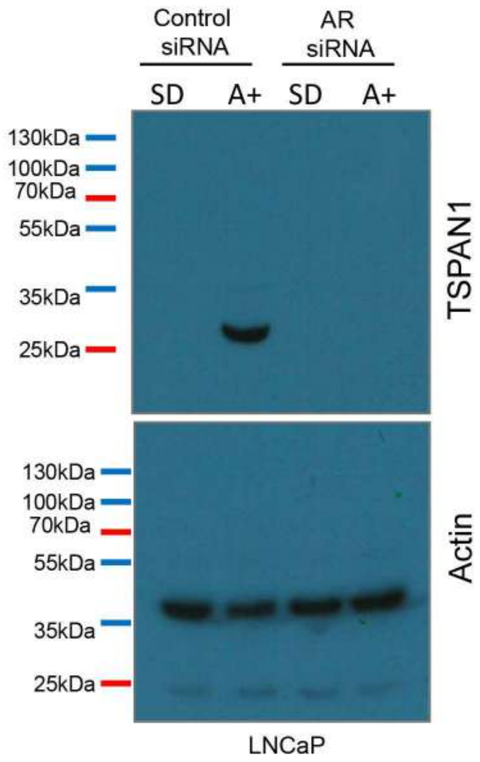

Figure 1F

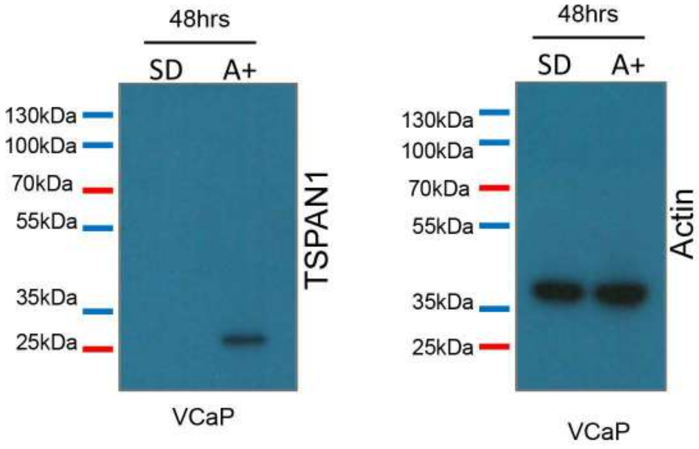

Figure 3C

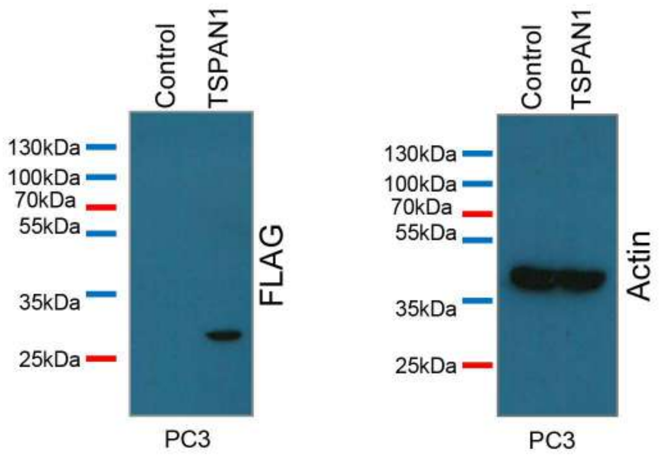

Figure 3E

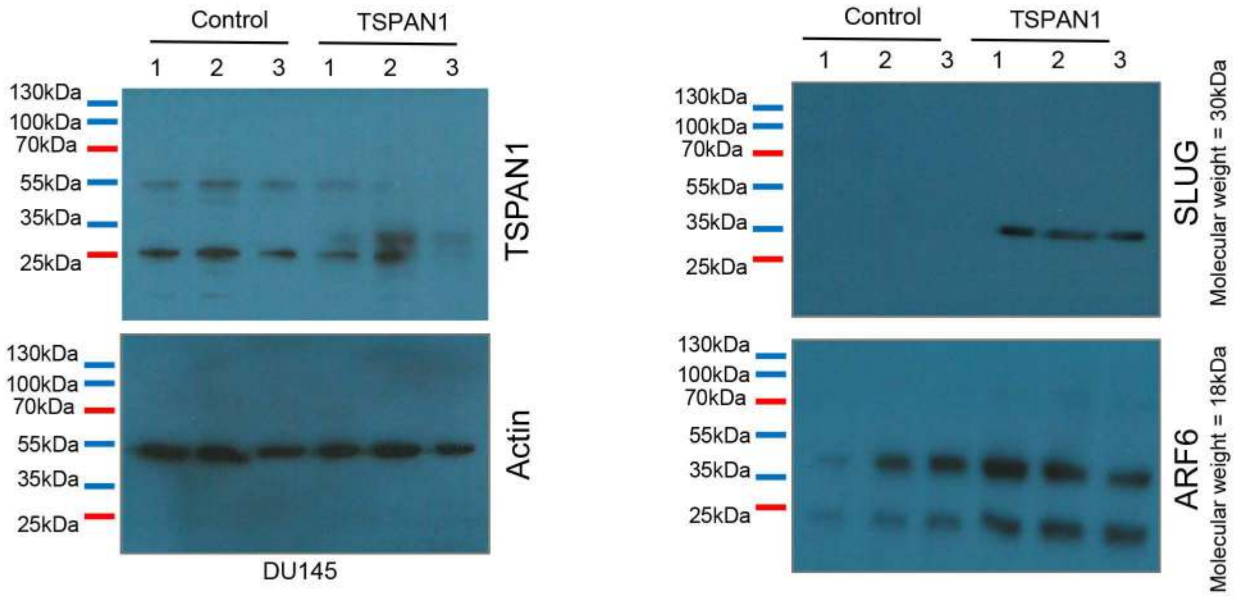

Figure 4A

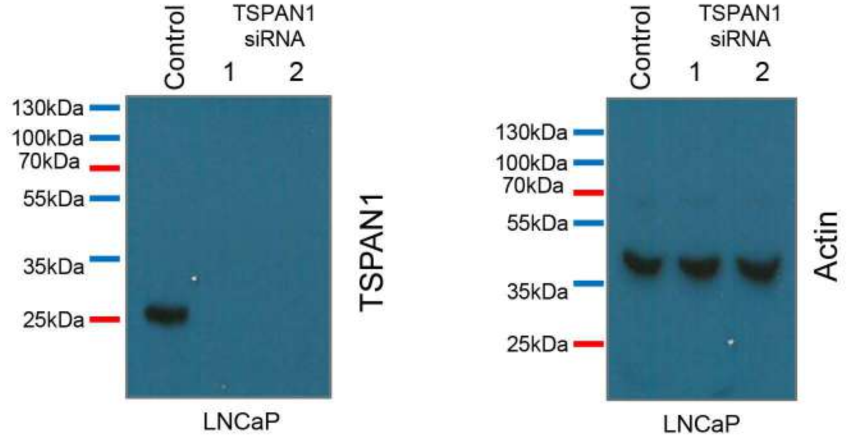

Figure 4C

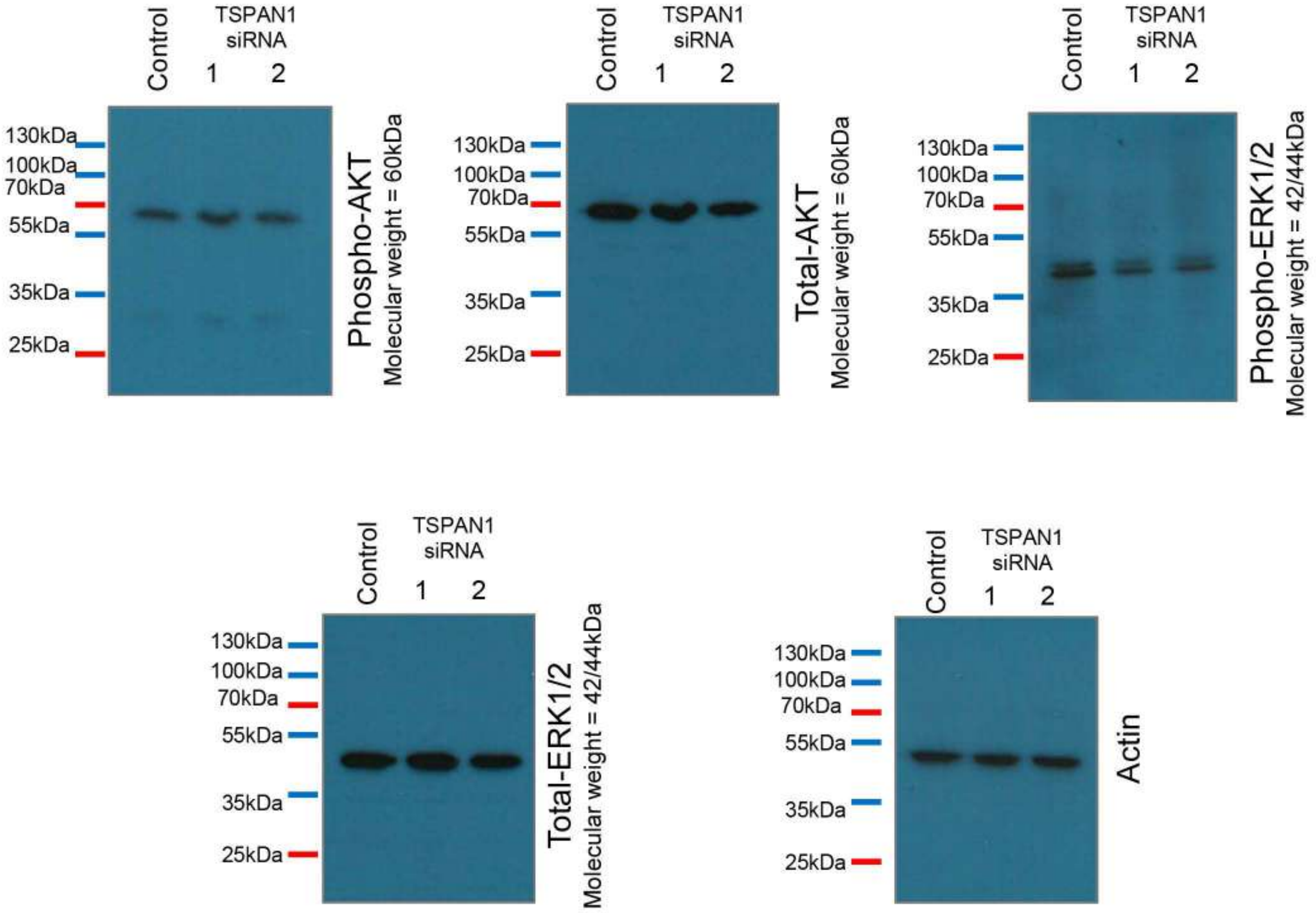

Supplementary Table 1: Meta-analysis of *TSPAN1* expression in prostate cancer

1012 samples

	**Grasso et al. 2012**	**Taylor et al. 2010**	**Singh et al. 2002**
	Prostate carcinoma v normal	Prostate carcinoma v normal	Prostate carcinoma v normal
	122 samples	185 samples	102 samples
**Fold Change**	2.214	1.844	8.438
**p value**	1.31E-06	1.72E-07	3.98E-08
	**Yu et al. 2004**	**Vanaja et al. 2003**	**LaTulippe et al. 2002**
	112 samples	40 samples	35 samples
	Prostate carcinoma v normal	Prostate carcinoma v normal	Prostate carcinoma v normal
**Fold Change**	2.322	2.213	7.751
**p value**	2.55E-05	4.64E-05	0.123
	**Wallace et al. 2008**	**Arredouani et al. 2009**	**LaPointe et al. 2004**
	Prostate carcinoma v normal	Prostate carcinoma v normal	Prostate carcinoma v normal
	89 samples	21 samples	112 samples
**Fold Change**	2.276	1.612	1.59
**p value**	2.00E-03	0.049	4.19E-04
	**Luo et al. 2002**	**Holzbeierlein et al. 2004**	**Welsh et al. 2001**
	Prostate carcinoma v normal	Prostate carcinoma v normal	Prostate carcinoma v normal
	30 samples	54 samples	34 samples
**Fold Change**	1.41	1.366	3.971
**p value**	1.15E-01	2E-01	4.33E-05
	**Varambally et al. 2005**	**Liu et al. 2006**	
	19 samples	Prostate carcinoma v normal	
	Prostate carcinoma v normal	57 samples	
**Fold Change**	2.303	1.574	
**p value**	0.007	8.87E-04	

TSPAN1 significantly upregulated in 11/14 datasets

Supplementary Table 2: Basic clinical data for patient tissue samples used in TMA.

Samples used in this study were collected between January 1989 and June 2003 from the Freeman Hospital patients. All samples were first analysed by a trained pathologist before selection of cores for TMA construction was performed.

Gleason	4 n=1	5 n=1	6 n=5	7 n=11	8 n=17	9 n=24	10 n=7	uk n=11
Age at diagnosis	40-49 n=1	50-59 n=5	60-69 n=12	70-79 n=28	80-89 n=4			uk n=26
Stage	T1 n=16	T1B n=7	T2 n=11	T3 n=24	T3C n=3	T4 n=11	T4B n=1	uk n=4
PSA	<10 n=8	10-19 n=11	20-29 n=4	30-49 n=10	50-99 n=6	100-200 n=4	<200 n=4	Uk n=30

Supplementary Table 3: Real-time PCR screen for 72 cell motility genes in DU145 TSPAN1 stable cell line

10 genes with significant changes:

**TSPAN1**	**RQ**	**SE**
Control	1.00	0.00
TSPAN1	6.62	2.16

**rac2**	**RQ**	**SE**
Control	1	
TSPAN1	2.01	0.19

**actn1**	**RQ**	**SE**
control	1.00	
TSPAN1	1.95	0.20

**ARF6**	**RQ**	**SE**
control	1.00	
TSPAN1	2.04	0.39

**DIAPH1**	**RQ**	**SE**
control	1.00	
TSPAN1	2.28	0.27

**ICAM1**	**RQ**	**SE**
control	1.00	
TSPAN1	2.00	0.28

**SNAI2/Slug**	**RQ**	**SE**
control	1.00	
TSPAN1	3.27	0.40

**actn3**	**RQ**	**SE**
control	1.00	
TSPAN1	0.65	0.11

**cav1**	**RQ**	**SE**
Control	1.000	
TSPAN1	0.500	0.05

**ptk2b**	**RQ**	**SE**
Control	1.000	
TSPAN1	0.510	0.03

**pxn**	**RQ**	**SE**
Control	1	
TSPAN1	0.36	0.030

67 genes with no change:

wasf2	capn1	stat3
fgf2	msn	baiap2
vegfa	myl9	plcg1
pld1	rock1	svil
myh9	ptk2	rnd3
itgb1	tln1	rdx
wipf1	ezr	crk
mapk1	vasp	rac1
igfr1	pak4	limk1
egfr	pfn1	pak1
met	tmp2	twist2
plaur	arhgdia	snai1
itgb3	ptpn1	vim
itgb2	akt1	cdh1
rac2	actn4	cdh2
prkca	actr2	
capn2	wasf1	
mmp14	fap	
ptk2	cfl1	
pten	pik3ca	
csf1	actr3	
enah	sh3pxd2a	
ilk	cttn	
vcl	mylk	
rasa1	cdc42	
bcar1	egf	

Supplementary Table 4: Differential analysis of somatic copy number and gene expression in neuroendocrine prostate cancer

Beltran et al. 2016

	% CRPC-NE deletion	% CRPC-Adeno deletion
TSPAN1	0.023255814	0.037735849
ARF6	0.093023256	0
SLUG	0.069767442	0

	% CRPC-NE amplification	% CRPC-Adeno amplification
TSPAN1	0.279069767	0.283018868
ARF6	0.325581395	0.377358491
SLUG	0.372093023	0.58490566

Supplementary Table 5: Real-time PCR primer sequences

	Forward	Reverse
**TSPAN1**	GAAAGGGCTCAAGTGCTGTG	TCATTGGCTGTGTTGGTGAC
**rac2**	GTTCCCCTTCCGTTTCCTC	ATCTCCCACCACCACACACT
**actn1**	GCTCCTGTGGTGTCAGAGAA	GCTTCCCGTAGTCAATCAGC
**ARF6**	CGCCATAATCCTCATCTTCG	TCATAGAGTCCGTCCCCTGA
**DIAPH1**	AGTCCCTTCGTGTGTCTCTCA	GGCTATCGTAACTCCCAGCA
**ICAM1**	CACTCTGGCTCCTTCACCAT	*AACCACTGACTCCCCTTCCT*
**SNAI2/Slug**	TGTCATACCACAACCAGAGA	CTTGGAGGAGGTGTCAGAT
**actn3**	CAGAGGAAGACAGCACCGTA	CTTTCGCAGTTTGGCGTAGT
**cav1**	ACTGGTTTTACCGCTTGCTG	GGAATAGACACGGCTGATGC
**ptk2b**	ATCATCACCTCCATCCTGCT	TCATACCTCCACTCGGCTTC
**pxn**	GGCTCTCTCATCATCCATCC	TTCAGGACCACATCTTCACG
**Actin**	CATCGAGCACGGCATCGTCA	TAGCACAGCCTGGATAGCAAC
**GAPDH**	AAC AGC GAC ACC CAT CCT C	CAT ACC AGG AAA TGA GCT TGA CAA
**Btubulin**	CTTCGGCCAGATCTTCAGAC	AGAGAGTGGGTCAGCTGGAA

Supplementary Table 6: Concentration of antibodies used

Antibody	Supplier	Product number	Concentration for WB	Concentration for IHC
TSPAN1	Sigma-Aldrich	HPA011909	1 to 2000	1 to 1500
AR	BD Bioscience	554226	0.5 µg/ml	
p21 Waf1/Cip1	Cell Signaling	2947	1 to 1000	
Slug	Cell Signaling	9585	1 to 1000	
p44/42 MAPK	Cell Signaling	9106	1 to 250	
Total ERK2	Santa Cruz	sc-1647	0.2 µg/ml	
pAKT	Sigma-Aldrich	SAB4300042	1 to 1000	
Total AKT	Santa Cruz	sc-8312	0.2 µg/ml	
ARF6	Cell Signaling	5740	1 to 1000	
Flag	Sigma-Aldrich	F3165	1 to 2000	
Actin	Sigma-Aldrich	A2668	1 to 2000	
normal rabbit IgG	Jackson labs	711-035-152	1 to 2000	
normal mouse IgG	Jackson labs	715-036-150	1 to 2000	
